# Bovine Leukemia Virus as a One Health Challenge in Nutrition, Food Security, and Public Health: A Narrative Review

**DOI:** 10.1016/j.tjnut.2026.101654

**Published:** 2026-06-06

**Authors:** Tanner F Scull, Oscar J Benitez

**Affiliations:** 1 Department of Veterinary Sciences, Davis College of Agricultural Sciences and Natural Resources, Texas Tech University, Lubbock, TX, United States; 2 School of Veterinary Medicine, Texas Tech University, Amarillo, TX, United States

**Keywords:** bovine leukemia virus, One Health, dairy production, milk composition, nutrition, food security, food systems, sustainability, public health

## Abstract

Bovine leukemia virus (BLV) is among the most prevalent infectious diseases of dairy cattle, with herd-level infection rates exceeding 80% in many regions worldwide. Although only a minority of infected animals develop clinical enzootic bovine leukosis, subclinical effects are widespread and extend beyond animal health. This narrative review examines BLV through a One Health lens, emphasizing its nutritional, food security, and public health dimensions. Relevant literature was identified through targeted searches of major scientific databases using terms related to BLV, milk composition, nutrition, food security, and One Health. Evidence suggests that BLV infection reduces milk yield and alters composition, particularly in cows with high proviral load. Decreases in fat, protein, and bioactive components diminish milk’s caloric density and functional quality, potentially lowering consumers’ dietary intake of essential fatty acids, fat-soluble vitamins, and immunomodulatory proteins. Increased susceptibility to mastitis due to BLV-associated immune dysfunction further exacerbates nutrient losses, compounding inefficiencies in milk production. Collectively, these changes undermine dairy’s nutritional value, especially for populations that rely heavily on milk as a staple food, including young children, low-income households, and communities in low- and middle-income countries where dairy products may provide important dietary energy, protein, and micronutrients. At the systems level, BLV contributes to economic inefficiency through reduced herd productivity. These losses aggregate into a significant drag on dairy supply, with implications for affordability, accessibility, and environmental sustainability. Although definitive evidence of zoonotic transmission is lacking, recurring detection of BLV deoxyribonucleic acid, proteins, and antibodies in human tissue raises questions regarding potential public health risks. Taken together, BLV represents an underappreciated One Health challenge, encompassing animal productivity, human nutrition, economic access, and possible zoonotic implications. Future research should quantify BLV’s economic and nutritional impacts on consumers and clarify risks to human health to support interventions aligned with animal health, food security, and sustainable nutrition.

## Introduction

Bovine leukemia virus (BLV) is one of the most widespread viral infections in dairy cattle worldwide, with herd-level prevalence exceeding 80% in major dairy-producing countries such as the United States and Canada, and similarly high rates reported in many other regions across the globe [1,2]. Despite its ubiquity, the broader One Health implications of BLV remain incompletely characterized, particularly with respect to nutrition, food security, and public health. This discrepancy is largely due to the fact that only a small percentage (<5%) of infected cattle develop enzootic bovine leukemia, a fatal lymphoproliferative disease [3]. However, the vast majority of infected animals experience subclinical effects, which, although not immediately apparent, have significant consequences for dairy productivity and animal health. Subclinical impacts include reduced milk yield, altered composition, immune dysfunction, increased secondary infection, and higher culling rates [[4], [5], [6], [7], [8]]. Collectively, these lead to economic losses and inefficiencies in milk production, yet BLV remains an underresearched and unappreciated issue in One Health [9].

The recent rise of the One Health approach has encouraged the scientific community to examine health challenges through a diverse, transdisciplinary lens, enabling the reexamination of long-standing issues from a multidimensional perspective. This framework acknowledges the interconnectedness of human, animal, and environmental health, and the influence of human activity on each [10]. Within this One Health framework, BLV remains a largely overlooked challenge in nutrition, food security, and public health. This virus not only affects animal health, milk production, and the economics of the dairy industry but also raises concerns over human nutrition and zoonotic potential. Additionally, environmental factors, including seasonal activity of hematophagous insects and climate-related changes in vector ecology, may contribute to BLV transmission and further complicate disease control [[11], [12], [13]].

Dairy production plays a vital role in global food systems, providing important macronutrients and micronutrients, including protein, lipids, calcium, and vitamins to millions of people worldwide. In many regions, milk and dairy products are a cornerstone of the human diet, contributing significantly to food security and nutritional health [14]. The cow–human interface, particularly through direct consumption of milk and dairy products, has profound implications for public health. However, as dairy production becomes increasingly industrialized, infectious diseases such as BLV emerge as critical concerns. By affecting both milk yield and quality, BLV threatens not only animal health but also nutrition and food security.

This narrative review examines BLV through a One Health lens, exploring its impact on nutrition, food security, and public health. Although BLV has been extensively studied from a virological and animal health perspective, prior reviews have largely emphasized viral biology, transmission, and clinical outcomes in cattle, with more limited attention to downstream effects on food quality, food systems, and human nutrition. In this review, we analyze key factors, including global prevalence, risk factors, changes in milk composition, and economic burden, because these factors represent key pathways through which BLV may influence nutrition, food security, and public health within a One Health framework.

This review was conducted as a narrative synthesis of interdisciplinary literature to examine BLV within a One Health framework. Relevant sources were identified through targeted searches using Google Scholar, PubMed, and Web of Science, using combinations of keywords such as “bovine leukemia virus,” “enzootic bovine leukosis,” “milk composition,” “nutrition,” “food security,” “food systems,” and “One Health.” Additional sources were identified through manual review of reference lists from key articles and reports from international organizations. Peer-reviewed original research articles, reviews, and relevant policy or surveillance reports were considered. Literature was selected to support a conceptual integration of animal health, nutrition, food security, environmental sustainability, and public health rather than to provide an exhaustive or systematic evaluation of all available studies.

## The One Health Framework in Nutrition and Food Systems

**FIGURE 1:**
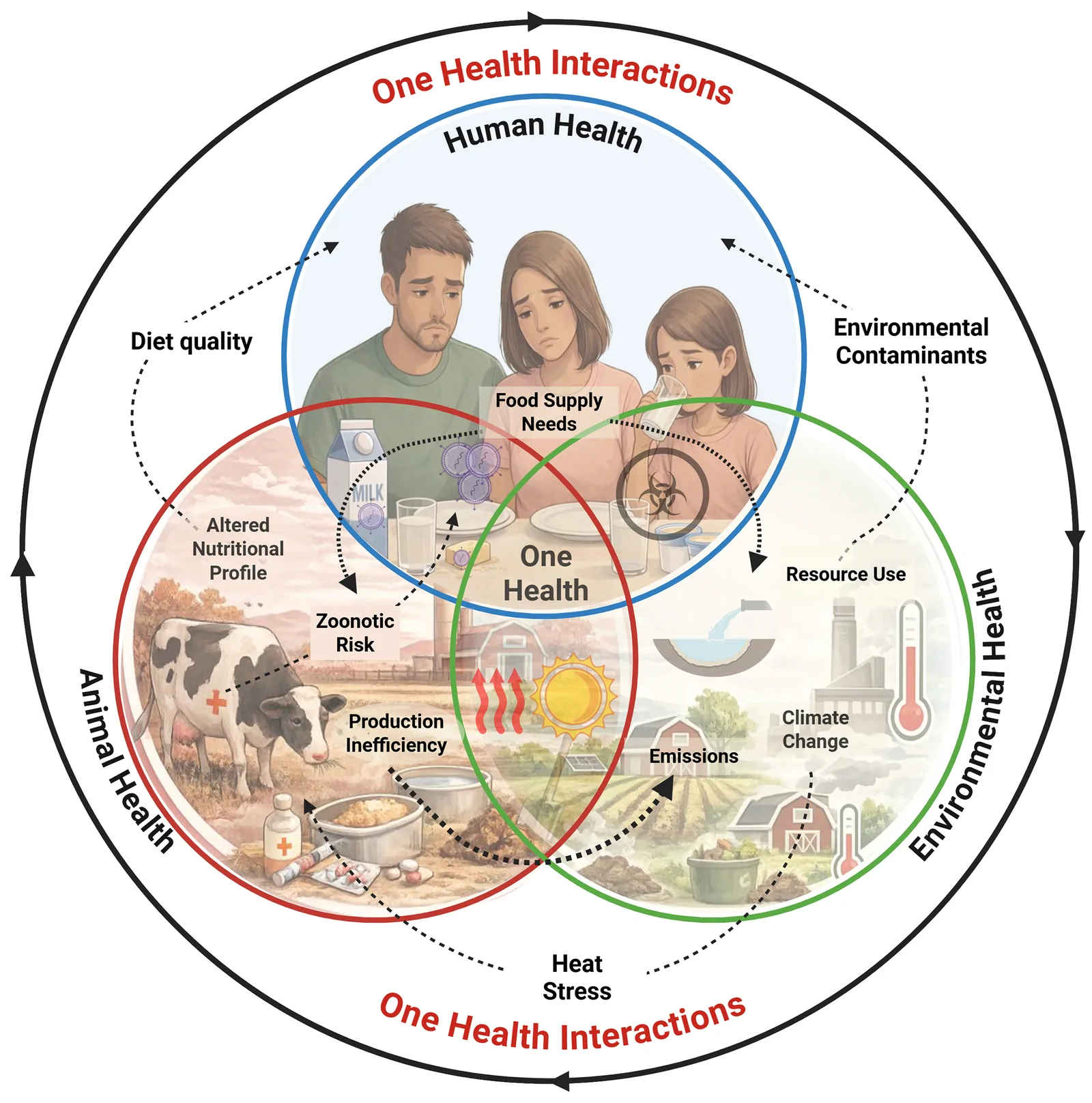
Conceptual illustration of the One Health framework applied to the role of BLV in food systems. BLV, bovine leukemia virus.

The One Health framework recognizes the interconnectedness of animal health, human health, and environmental health, emphasizing that outcomes in 1 domain can have cascading effects across others (Figure 1). It has been widely adopted by organizations such as the WHO, the FAO, and the World Organization for Animal Health [15]. Traditional One Health reviews have largely focused on acute and readily observable challenges, including emerging zoonotic diseases, antimicrobial resistance, food safety, and environmental drivers of infectious disease [10,16,17]. In contrast, the application of One Health frameworks to nutrition, food quality, and food security has been comparatively limited, despite the central role of animal-derived foods in global diets.

Where One Health frameworks have been applied in nutrition-related contexts, they have most often taken the form of food systems-oriented analyses, rather than direct evaluations of nutrient quality or dietary intake. This body of work has emphasized food safety across animal-to-human supply chains, the food system consequences of zoonotic disease outbreaks, antimicrobial use in food-producing animals, and sustainability-focused assessments that integrate livestock production with environmental and human health outcomes [[18], [19], [20]]. One Health perspectives have also been used to examine sustainable diets, linking nutritional considerations with environmental resource use and animal production systems [21]. Together, these studies demonstrate the relevance of One Health to nutrition, while also illustrating that nutrition is typically considered alongside broader food system, environmental, and public health outcomes.

Taken together, these applications illustrate the value of the One Health framework for examining interconnections among animal health, environmental conditions, and human outcomes within food systems. Rather than prioritizing a single domain, One Health provides a structure for considering how changes in livestock health may influence nutritional quality, food system efficiency, environmental sustainability, and public health simultaneously. Although prior One Health work in food systems has largely emphasized production, safety, and sustainability, comparatively less attention has been given to how specific livestock diseases shape the nutritional quality of foods derived from affected animals. BLV is particularly well suited to examination through a One Health lens because it is widespread and operates primarily through food system pathways. As such, BLV highlights how livestock health conditions can generate cascading effects across animal production, nutrition, and food security. In this review, the One Health perspective is used to situate BLV within this broader systems context and to guide the synthesis of evidence across animal health, nutrition, food security, and public health.

## Epidemiology of BLV

The epidemiology of BLV is driven by complex interactions between viral biology, host immunity, management practices, and environmental context. BLV is a retrovirus that infects and integrates its genome into B-cells. Throughout the infection, cell-free virus is rarely found, limiting opportunities for direct viral transmission [3]. Thus, BLV is transmitted primarily through the transfer of infected lymphocytes. Horizontal transmission is the dominant route and typically occurs via blood-contaminated equipment such as needles and dehorners. Close-contact practices, including milking and feeding, can facilitate the exchange of lymphocyte-containing bodily fluids, and the reuse of rectal-palpation sleeves presents additional risk [22]. Transmission by biting insects such as horseflies has also been reported [23]. Vertical transmission occurs in utero or through colostrum, with risk highest when dams carry a high proviral load [[24], [25], [26]].

Several management and biosecurity factors further amplify the likelihood of BLV spread within and between herds. Because BLV resides in infected lymphocytes, even trace amounts of residual blood on reused or improperly disinfected equipment can initiate infection. Practices that create opportunities for iatrogenic blood contact, such as vaccination, dehorning, ear-tagging, rectal palpation, and artificial insemination, are consistently associated with increased transmission and higher proviral load [[27], [28], [29], [30], [31], [32]]. Inadequate biosecurity protocols also play a central role in facilitating BLV transmission. Farms that do not implement closed-herd policies or that purchase replacement animals from untested herds are at a significantly greater risk of introducing BLV into their operations. Failure to isolate or test new arrivals increases the risk of introducing asymptomatic carriers [[33], [34], [35]].

The influence of production system on BLV risk remains insufficiently defined, an important gap as interest grows in pasture-based dairy systems and precision grazing technologies [36,37]. Argentina provides a useful example because dairy production remains largely pasture based, yet BLV remains endemic in dairy cattle there [38,39]. In contrast, pasture-based or grazing-oriented systems in New Zealand and parts of Australia have achieved BLV eradication or very low prevalence through coordinated surveillance and control programs [40,41]. In some settings, grazing or loose-housing systems may increase contact opportunities among infected and susceptible animals; for example, a study of Egyptian dairy cattle found higher odds of BLV seropositivity among grazing cattle in loose-housing systems [42]. Overall, these examples highlight an important knowledge gap: as dairy production systems continue to shift, additional research is needed to determine how pasture access and related variables interact to influence BLV transmission.

Environmental stressors, including overcrowding, heat stress, and nutritional deficiencies, may compromise immune function, which may increase susceptibility to BLV infection and promote viral replication and proviral accumulation [43]. Climate change is likely to further complicate the epidemiology of BLV. Rising global temperatures may extend the active seasons and geographic ranges of hematophagous insect populations, potentially increasing opportunities for vector-borne transmission [[11], [12], [13],^44^]. Additionally, heat stress, already a major concern in dairy operations, may become more prevalent, compounding its effects on both immunity and productivity [43]. As such, climate-related changes in animal physiology, stress levels, and vector ecology should be closely monitored for their potential to shift BLV dynamics, even in currently stable regions.

Framing BLV control solely as a veterinary issue overlooks its sustainability cobenefits. By lowering culling rates and secondary infections, effective control can reduce greenhouse gas emissions per unit of milk [45], limit manure output, and decrease antimicrobial use—issues central to both One Health and sustainable food systems [46,47]. Healthy, longer-lived cows thereby align animal welfare with economic and environmental sustainability.

## Global Prevalence

BLV remains one of the most widespread infectious diseases of cattle worldwide, though prevalence varies dramatically across regions. In countries with strong control measures, particularly in the European Union, systematic control and surveillance programs have successfully eliminated BLV. Finland, for example, initiated official control measures in 1966 and eradicated EBL from mainland Finland in 1996 through a nationwide test-and-slaughter program supported by herd restrictions, systematic surveillance, detailed animal recordkeeping, and government compensation for culled animals [48,49]. These successes demonstrate that eradication is technically feasible when robust infrastructure, funding, and compliance are in place.

In stark contrast, both North and South American countries face widespread endemic infection. In the United States, over 89% of dairy herds and >40% of individual cows are infected, with prevalence reaching >95% in some large commercial operations [2,50]. Canada lacks a national prevalence report, but available regional studies consistently show similarly high herd-level infection rates [1,51]. In South America, BLV is widespread, though prevalence estimates vary considerably by country. Some of the highest herd-level rates have been reported in Argentina (∼91%) and Colombia (∼92%), according to recent epidemiological surveys [[52], [53], [54]].

In Asia, BLV prevalence is also pronounced but highly variable by country. In China, for example, a meta-analysis estimated a pooled BLV prevalence of 10.0% among cattle, although prevalence varied widely across regions [55]. In Japan, surveys of dairy farms report that ∼79% of farms are infected; among infected farms, the proportion of seropositive cattle varies considerably, with median within-herd seroprevalence around 48%, and some herds exceeding 60% to 70% [27]. India, Thailand, and other developing countries in Asia lack comprehensive BLV surveillance data; however, seroprevalence studies indicate a substantial presence of the virus with marked regional variability [54,56,57].

## Nutritional Implications of BLV

Milk and dairy products are consumed globally, with high daily intake in many countries. In 2018, global per-capita availability averaged ∼227 g/d [58], underscoring milk’s central role in the human diet. Milk is a nutrient-dense food [59] that enhances the overall nutritional quality of diets in which it is included [60], making it a consequential food in terms of public health [61,62]. Its composition, rich in proteins, lipids, minerals, and vitamins, makes it an essential source of nutrition [63,64].

The chemical composition of milk varies with animal-level factors such as environment, nutritional status, genetics, and stage of lactation [65]. In addition to these natural sources of variation, research indicates that BLV infection can also alter milk composition [66], potentially affecting its nutritional value. Lipids represent a significant fraction of milk content, typically around 4%, and serve multiple critical functions in human nutrition. They provide a concentrated source of energy, essential fatty acids, fat-soluble vitamins, and lipid-derived biomolecules such as phospholipids [67,68]. Multiple studies have shown that cows infected with BLV produce less milk fat than their uninfected counterparts [66,[69], [70], [71]]. More recent herd-level work highlights that this reduction is concentrated among cows with high proviral loads, which consistently exhibit lower milk, fat, and protein yields [71].

A decline in milk fat content has several potential nutritional consequences. Because fat is a major contributor to the caloric value of milk, reduced fat levels may lower the overall energy provided by dairy products, an effect particularly relevant in populations that rely heavily on milk for dietary energy. The concern is especially acute for young children, for whom adequate fat intake is essential for growth, development, and overall health [72]. Milk fat contains bioactive fatty acids, including butyrate and other short- and medium-chain fatty acids, which contribute to its nutritional and functional properties [73,74]. In addition, milk is a good source of oleic acid [75], a MUFA associated with improved lipid metabolism and cardiovascular health [76,77]. With a decrease in overall milk fat production, consumers could be receiving lower amounts of these important biomolecules.

Fat-soluble vitamins A, D, and E are closely linked to milk fat content. Milk is a major dietary source of vitamin A, which is essential for vision, cell differentiation, embryogenesis, and reproduction [78]. A decrease in fat concentration in milk may lead to lower levels of these vitamins, potentially contributing to the risk of deficiencies, particularly in populations where dairy serves as a major dietary source of these nutrients [79].

Milk provides about 3% protein by weight, supplying indispensable amino acids in highly digestible forms. These proteins are broadly categorized into caseins and whey proteins, both of which are considered high-quality due to their complete amino acid profiles [80]. In addition to their nutritional value, milk proteins and peptides such as *β*-lactoglobulin and caseins retain bioactivity after consumption, contributing to digestion, immune regulation, and metabolic health [81]. BLV infection has been associated with reduced protein yield in dairy cows [66,71]. Beyond lowering total protein content, BLV may impair the synthesis of specific bioactive proteins. *In vitro* evidence shows that BLV disrupts mammary epithelial cell function, reducing casein production [82]. This is particularly important because casein accounts for ∼80% of total milk protein and facilitates the binding and absorption of calcium and phosphorus, enhancing bone health [83,84]. Caseins also serve as precursors to bioactive peptides with antioxidant, immunomodulatory, and antihypertensive effects [85,86]. Thus, a reduction in casein not only decreases total protein intake but may also compromise mineral bioavailability and the generation of health-promoting peptides.

Recent evidence further shows that BLV-infected cows produce milk with significantly lower levels of lactoferrin, lactoperoxidase, and α-lactalbumin [87]. Lactoferrin, an iron-binding glycoprotein, regulates iron homeostasis, promotes the growth of beneficial bacteria, and exhibits both antimicrobial and immunomodulatory properties [88]. Lactoperoxidase contributes to milk’s natural defense mechanisms by generating antimicrobial compounds through thiocyanate oxidation [89]. α-lactalbumin, which makes up about 17% of whey protein, is central to lactose synthesis and can also provide antimicrobial peptides with immunomodulatory activity on digestion [90]. A decrease in these proteins could compromise the natural protective properties of milk, reducing its overall functional benefits for consumers. Producing nutritionally diminished milk with unchanged inputs represents a dual inefficiency; more resources are consumed to yield milk of lower nutritional value. This undermines both consumer nutrition and the sustainability of dairy production [4,91,92].

One of the most deleterious effects of BLV infection is immune dysfunction, which increases cattle’s susceptibility to secondary diseases [4,93]. Mastitis—the inflammation of the mammary gland, typically caused by bacterial infection—is among the most common and well-studied secondary conditions associated with BLV. It is a major global challenge to the dairy industry due to treatment costs, mortality, and decreased milk production [94]. Across cohort studies, BLV is associated with a significantly higher risk of subclinical mastitis, along with higher odds of greater clinical severity among mastitis cases [7,[95], [96], [97]]. Mastitis in dairy cattle induces profound changes in milk composition through inflammatory responses and proteolytic degradation of key nutrients. Multiple studies document significant reductions in milk fat, protein, and lactose, along with shifts in fatty acid and bioactive protein profiles. Reported reductions in fat average 15% to 40%, affecting both saturated and unsaturated fatty acids, whereas protein losses range from 10% to 30% and include marked decreases in casein. Lactose levels fall by 5% to 15%, reflecting impaired synthesis by damaged mammary epithelial cells [[98], [99], [100], [101]]. Proteolytic degradation exacerbates these nutrient losses: *Streptococcus agalactiae* can degrade ≤75% of caseins and 21% of whey proteins *in vitro* [102], whereas *Staphylococcus aureus* infections cause similar reductions, significantly compromising nutritional integrity and functionality [103].

Taken together, the evidence indicates that BLV infection and its associated immune dysfunction can lead to detrimental changes in milk composition, both directly and through secondary infections such as mastitis. These changes consist of reductions in fat, protein, lactose, and bioactive components, compromising milk’s nutritional density, functional quality, and potential health benefits (Table 1). Given the global reliance on dairy products as a key source of energy, essential fatty acids, amino acids, and micronutrients, these compositional shifts carry important public health implications, particularly in populations where milk is a dietary staple. Nutrition management may modify these outcomes, as dietary strategies and feed additives that influence immune function, oxidative balance, and udder health could affect the severity of BLV-associated secondary outcomes, including mastitis and milk-composition losses [104,105]. Future research should prioritize longitudinal studies to better quantify these impacts and inform strategies to mitigate BLV-associated reductions in milk quality, nutritional value, and production efficiency at the herd and industry levels, including approaches that address secondary outcomes such as mastitis.

**TABLE 1:** Nutritional implications of BLV and mastitis on milk composition

Milk component	Effect in BLV	Effect in mastitis	Nutritional implication	References
Milk fat and fatty acids	Among cows with high proviral load or persistent lymphocytosis, BLV is associated with lower milk fat yields and altered fatty acid profiles (e.g., decreases in short-chain and MUFAs).	In mastitis, total fat may decline by 15%–40%, and the balance of saturated vs. unsaturated fats can shift.	Fat contributes substantially to milk’s caloric density; short-chain fatty acids also support gut health and immune function.	[[66], [67], [68], [69], [70], [71], [72],^75^]
Fat-soluble vitamins (A, D, E)	Reduced fat transport may lead to lower concentrations of vitamins A, D, and E in milk.	Mastitis-associated fat loss may reduce these vitamin levels.	Vitamins A, D, and E are critical for vision, immune function, and antioxidant defense.	[78,79]
Protein (total, casein, whey)	BLV (especially in high PVL cows) is linked to reduced total protein yield, impaired casein synthesis, and lower levels of whey proteins.	Mastitis often causes 10%–30% reductions in total protein, with proteolysis of caseins and whey proteins.	Milk proteins supply indispensable amino acids; caseins assist mineral absorption and bioactive peptides support immunity.	[66,71,72,[82], [83], [84], [85], [86],^102^,^103^]
Bioactive proteins (lactoferrin, lactoperoxidase, α-lactalbumin)	BLV-positive cows can exhibit lower levels of lactoferrin, lactoperoxidase, and α-lactalbumin; possible downregulation by BLV miRNAs.	Mastitis triggers proteolysis and enzymatic degradation of these proteins.	These proteins play antimicrobial and immunomodulatory roles in milk.	[[87], [88], [89], [90],^102^,^103^]
Lactose	BLV may indirectly affect lactose via increased risk of mastitis; direct effects are not well documented.	Milk lactose often declines by ∼5%–15% during mastitis, due to damage to secretory epithelium.	Lactose is a principal carbohydrate in milk, essential for energy (especially in infants).	[[98], [99], [100], [101]]

Abbreviations: BLV, bovine leukemia virus; miRNA, microRNA; PVL, proviral load.

## Food Security

**FIGURE 2:**
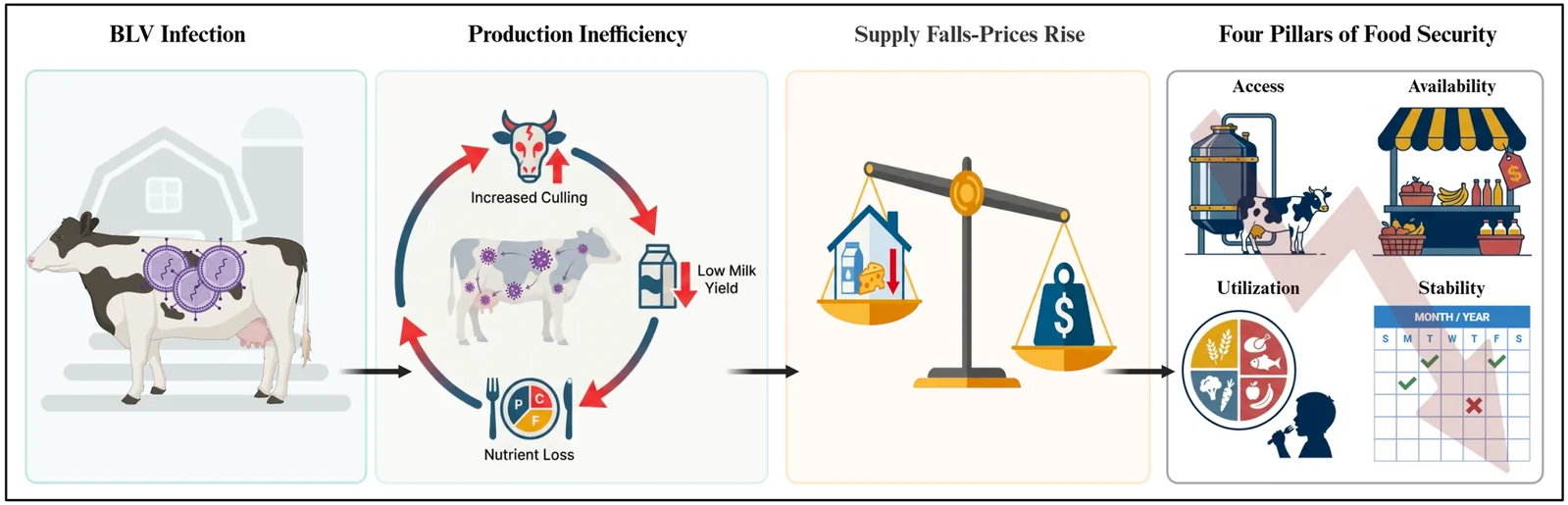
Graphic visualizing food security outcomes of BLV-related production inefficiency. BLV, bovine leukemia virus.

The global prevalence of BLV may represent an underappreciated challenge to food security, particularly among populations for whom milk can be an important source of dietary energy and nutrients, including young children in low- and middle-income countries and low-income households with limited access to alternative sources of high-quality protein, lipids, calcium, and micronutrients [106,107]. Food security is defined as a condition “when all people, at all times, have physical, social and economic access to sufficient, safe and nutritious food that meets their dietary needs and food preferences for an active and healthy life” [108]. This concept has 4 dimensions: availability, access, utilization, and stability—all of which depend on the productivity of food systems, which BLV undermines (Figure 2).

BLV infection reduces milk yield, increases culling rates, and compromises reproductive efficiency, thereby lowering herd productivity. These production losses translate into reduced dairy availability and higher consumer costs, undermining food security by raising the cost barrier to access to affordable, nutrient-rich foods. Large-scale herd-level studies in Sweden and the United States reported 2.5% and 2.7% lower milk production, respectively, in BLV-positive herds compared with uninfected herds. The Swedish analysis included 14,424 dairy herds with ≥10 cows enrolled in the official milk recording scheme, with BLV status determined by bulk-milk ELISA; individual cows were not tested for BLV status in that study. The United States analysis tested 25,956 cows from 1006 herds in 20 states and included 976 herds with complete data in the final multivariable analysis [109,110]. Subsequent analyses in Michigan have shown similar reductions, along with shortened productive lifespan and higher culling rates [[111], [112], [113]].

Although these productivity losses may appear modest at the individual herd level, they aggregate to a substantial source of inefficiency in regional and global dairy output. Because BLV often remains subclinical, its impact is easily overlooked in policy planning, leading to limited investment in surveillance and control. Economic analyses estimate that BLV infection imposes an annual economic burden of about US $525 million (2003) on the United States dairy industry, largely due to reduced milk production, early culling, and impaired reproduction. This figure likely underrepresents the full cost, as it does not account for secondary expenses such as veterinary care and management inefficiencies [9,109]. These costs are often absorbed silently within production systems, as the slow, persistent nature of the disease can mask its cumulative impact. However, as prevalence levels remain high, exceeding 80% in many regions, these subclinical losses compound into a significant drag on national dairy efficiency and profitability.

This economic inefficiency has downstream effects on dairy market dynamics. Reduced milk supply not only weakens the economic sustainability of dairy operations but also constrains access to affordable dairy. Economic welfare models of other dairy diseases in the United States have shown that reduced milk production decreases consumer surplus, raises retail milk prices, and diminishes supply [114,115]. In low- and middle-income countries, such dynamics are particularly concerning. In Kenya, an increase in milk price was associated with reduced milk allocation to young children, with households substituting less nutritious alternatives and thereby risking child nutrition and development [107]. Similar dynamics are evident globally. A recent study of 38 low- and middle-income countries demonstrated that dairy tariffs are positively associated with child stunting probability, and reductions in dairy tariffs, which improved consumer access, were linked to measurable declines in child stunting [116]. These results are in line with broader economic analysis showing that high dairy prices and wealth disparities are among the strongest predictors of low dairy consumption in low- and middle-income countries [117]. Together, this evidence highlights the central role of dairy affordability in shaping child nutrition and growth outcomes.

Beyond economic inefficiency, BLV undermines environmental sustainability. Infected cows consume the same feed, water, and land while producing less milk, increasing greenhouse gas emissions and resource use per liter of milk produced. Because improving production efficiency is a recognized strategy to mitigate dairy-related emissions [45], BLV acts directly against sustainability goals. These inefficiencies compound at high prevalence, threatening both food security and sustainability.

Thus, BLV’s impact on herd productivity not only compromises the economic sustainability of dairy operations but also threatens equitable access to affordable dairy and contributes to environmental inefficiency. These dynamics underscore BLV’s broader implications for food security, nutritional equity, and the need for One Health–aligned policymaking.

## Zoonotic Risk

Reports of BLV DNA and antibodies detected in human tissues have prompted suggestions of zoonotic potential, a possibility strengthened by the virus’s phylogenetic and functional resemblance to human T-cell leukemia virus (HTLV). Both BLV and HTLV are oncogenic delta retroviruses that integrate into the host genome, establishing lifelong infections. They share conserved regulatory genes, including *tax*, which encodes the Tax protein, a regulator of viral gene expression, infectivity, and host cell proliferation [118,119]. Both viruses suppress host antiviral responses and employ mechanisms of latency, clonal expansion, and immune modulation to evade immune detection. These strategies collectively compromise the immune system, increasing susceptibility to secondary infections [120,121]. Globally, an estimated 5 to 10 million people are infected with HTLV-1, and roughly 5% of those individuals develop severe diseases such as adult T-cell leukemia/lymphoma [122]. Given these structural, genetic, and pathogenic parallels, growing attention has turned to whether BLV might act as a zoonotic agent, particularly in populations with high exposure risk, such as consumers of unpasteurized dairy products.

Over the past decade, several studies have reported BLV DNA, protein, and BLV reactive antibodies in human tissues, including lung [123], blood [[124], [125], [126]], and most notably breast cancer tissue [127], raising questions about a possible zoonotic role in oncogenesis. One of the most prominent investigations, a case-control study of 239 women by Buehring et al. [127] found BLV DNA in 59% of breast cancer tissues compared with 29% of normal breast tissues, suggesting a strong association between BLV and malignancy. More recently, a systematic review and meta-analysis of 12 similar case-control studies found that BLV infection is statistically associated with an increased risk of breast cancer, with a pooled odds ratio of 2.12, although the authors acknowledge the existence of conflicting evidence [128].

In parallel, an *in vitro* study demonstrated that BLV can infect a range of human cell lines, providing experimental support that the virus is capable of entering human cells and establishing proviral DNA. However, these experiments did not assess whether BLV undergoes productive replication, expresses functional viral proteins, or integrates stably into the host genome, key requirements for a persistent infection. Consequently, this evidence indicates cellular susceptibility but does not confirm the capacity for sustained or transmissible infection in humans [129]. Complementing this work, cationic amino acid transporter 1 (CAT1), a widely expressed surface protein encoded by *SLC7A1*, has been identified as a functional BLV receptor, providing a plausible molecular pathway for cross-species susceptibility [130]. Because CAT1 is conserved across mammalian species and expressed in diverse human tissues, this discovery strengthens the biological framework for how zoonotic infection could occur.

**FIGURE 3:**
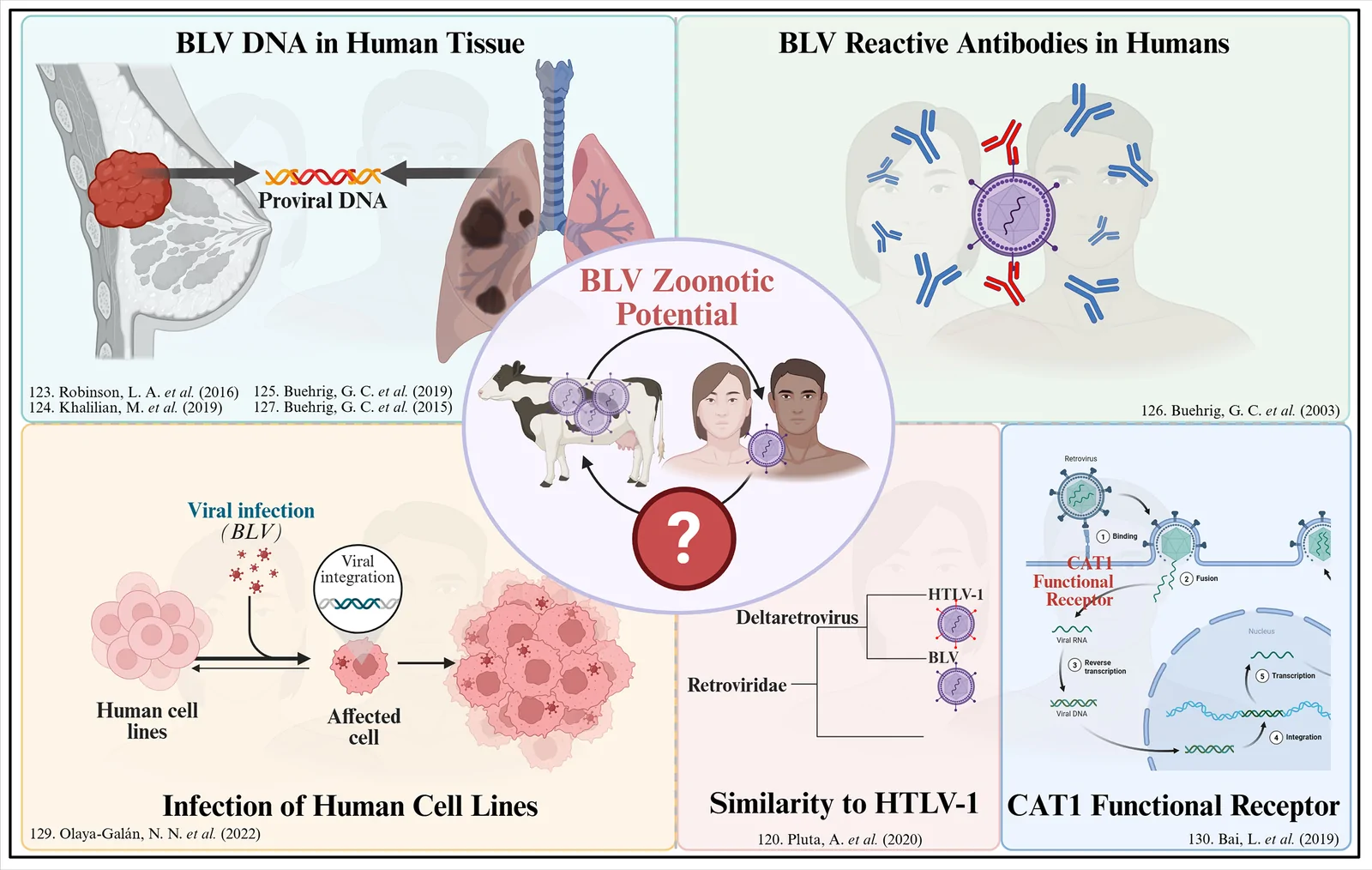
Graphic visualizing possible evidence of BLV zoonotic potential. BLV, bovine leukemia virus; CAT1, cationic amino acid transporter 1; HTLV-1, human T-cell leukemia virus type 1.

Although definitive evidence of zoonotic transmission is lacking, the observed similarities to HTLV, detection of BLV in human tissues, and demonstrated susceptibility of human cells *in vitro* justify further study (Figure 3). This body of evidence highlights ongoing uncertainty about BLV’s ability to cross-species barriers and its potential role in human disease. Each of these findings has limitations, and causality in human disease remains unproven. However, given the very high prevalence of BLV in cattle and the extent of human exposure through dairy products and occupational contact, even a low-probability zoonotic risk could have meaningful public health implications. From a One Health standpoint, precautionary measures such as continued BLV surveillance, rigorous pasteurization, and further research into human susceptibility remain prudent.

## Implications and Recommendations

Viewing BLV through a One Health framework highlights several priorities for future research. Additional studies are needed to more fully characterize how BLV infection influences milk quality, including changes in macronutrient composition and bioactive components, and to determine how these effects vary across infection status and proviral load. Beyond quantifying these impacts, future work should evaluate practical mitigation strategies that reduce BLV-associated losses in milk quality, animal health, and production efficiency. This should include assessing whether nutrition management and feed-additive strategies that support immune function, antioxidant status, or udder health modify BLV-associated secondary disease risk or milk-quality outcomes. Work is also needed to better quantify the efficiency costs associated with BLV, including impacts on production efficiency, economic outcomes, and resource use. In addition, investigation into opportunities for human exposure to BLV, and basic research further exploring a molecular basis for zoonotic potential, and the relevance of such findings to public health, would help clarify unresolved concerns surrounding zoonotic considerations. Integrating these lines of inquiry would support a more comprehensive understanding of how BLV operates across animal health, nutrition, and food systems.

From an epidemiological and policy perspective, the widespread and largely subclinical nature of BLV underscores the importance of improved monitoring and data integration, particularly the linkage of animal health, milk quality, and production data, rather than immediate intervention. Incorporating BLV status into existing herd health, milk quality, or surveillance frameworks could enhance understanding of its prevalence and production-level impacts while informing risk assessment efforts. Increased communication and education for producers regarding BLV transmission, management, and potential downstream consequences may also support informed decision-making without imposing additional regulatory burdens. Such approaches align with One Health principles by emphasizing data generation and awareness across animal health and food systems.

In conclusion, BLV exerts a silent but substantial toll on global dairy systems. Beyond reducing milk yield, BLV alters the nutritional profile of milk and increases susceptibility to secondary diseases such as mastitis. These biological impacts contribute to production inefficiencies that elevate costs and increase greenhouse gas emissions. These changes carry downstream effects for consumers: diminished nutritional quality, higher prices, and constrained access to dairy in vulnerable populations, undermining key dimensions of food security, including access, utilization, and stability. Although definitive evidence of zoonotic transmission is lacking, recurring detection of BLV in human tissues and its close parallels with HTLV highlight the importance of vigilance. Future work should prioritize large-scale studies to quantify BLV’s economic and nutritional consequences for consumers and refine our understanding of transmission dynamics. Addressing these questions will be essential for enabling effective control programs. Ultimately, BLV should be recognized not only as an animal health problem but as a One Health challenge that encompasses animal productivity, human nutrition, public health, and the sustainability of food systems worldwide.

## Author contributions

The authors’ responsibilities were as follows – TFS: conducted the literature search and drafted the manuscript; OJB: provided supervision, contributed to interpretation of the literature, and critically revised the manuscript; and all authors: conceived the review concept and scope and prepared figures and visualizations, reviewed and approved the final manuscript.

## Data availability

No new data were generated or analyzed in this study. Data sharing is not applicable to this article.

## Declaration of generative AI and AI-assisted technologies in the writing process

During the preparation of this work, the authors used ChatGPT to improve readability and language. After using this tool/service, the authors reviewed and edited the content as needed and take full responsibility for the content of the published article.

## Funding

The authors reported no funding received for this study.

## Conflict of interest

The authors report no conflicts of interest.
